# Beneficial Role of Bitter Melon Supplementation in Obesity and Related Complications in Metabolic Syndrome

**DOI:** 10.1155/2015/496169

**Published:** 2015-01-12

**Authors:** Md Ashraful Alam, Riaz Uddin, Nusrat Subhan, Md Mahbubur Rahman, Preeti Jain, Hasan Mahmud Reza

**Affiliations:** ^1^Department of Pharmaceutical Sciences, North South University, Dhaka 1229, Bangladesh; ^2^Department of Pharmacy, Stamford University Bangladesh, Dhaka 1217, Bangladesh; ^3^School of Biomedical Sciences, Charles Sturt University, Wagga Wagga, NSW 2678, Australia

## Abstract

Diabetes, obesity, and metabolic syndrome are becoming epidemic both in developed and developing countries in recent years. Complementary and alternative medicines have been used since ancient era for the treatment of diabetes and cardiovascular diseases. Bitter melon is widely used as vegetables in daily food in Bangladesh and several other countries in Asia. The fruits extract of bitter melon showed strong antioxidant and hypoglycemic activities in experimental condition both *in vivo* and *in vitro*. Recent scientific evaluation of this plant extracts also showed potential therapeutic benefit in diabetes and obesity related metabolic dysfunction in experimental animals and clinical studies. These beneficial effects are mediated probably by inducing lipid and fat metabolizing gene expression and increasing the function of AMPK and PPARs, and so forth. This review will thus focus on the recent findings on beneficial effect of *Momordica charantia* extracts on metabolic syndrome and discuss its potential mechanism of actions.

## 1. Introduction 

The prevalence of obesity is increasing at an alarming rate and has become one of the world's most serious public health problems. It has been estimated that 58% of world population will become obese by 2030 [1]. Global survey data also indicate that the prevalence of both male and female overweight and obesity varies by region and has rapidly increased in recent years [2, 3]. Elements that cause obesity involve metabolism, several genetic factors, diet, and physical activity, as well as the sociocultural surroundings that characterize the modern day living [4]. Recent evidences suggest that high fat diet, which is also characteristic of cafeteria type diet, as well as sedentary life style are two contributory factors for increased trends of obese people among the nations [5]. However, genetic factors contribute to the variation of adiposity in approximately 40–70% of a population [6]. These genetic factors thus explain the failure of exercise and dietary regime to bring about long-term weight loss in some individuals. Obesity can be defined as increased energy intake than energy expenditure which ultimately results in fat deposition and weight gain. According to guidelines from the World Health Organization (WHO), overweight in adults is defined by body mass index (BMI) of 25.0 to 29.9, and obesity is defined by a BMI of 30.0 or higher [7]. High body fat increases the risk of several diseases such as diabetes, hyperlipidemia, and hypertension, which may lead to arteriosclerotic disease and metabolic syndrome [8]. Consequently, obesity and related cardiovascular complications are also increasing alarmingly both in developed and developing countries. Adipocyte dysfunction and inflammation contribute to the various complications associated with obesity. Recently, adipose tissues are considered as an endocrine organ which secretes numerous fat and glucose regulating hormones, adipokines, and cytokines like adiponectin, leptin, and tumor necrosis factor-*α* (TNF-*α*) [9, 10]. Increased concentration and expression of TNF-*α*, interleukin-6 (IL-6), and monocyte chemoattractant protein-1 (MCP-1) are evident in adipocyte dysfunction and insulin resistance [11]. Furthermore, inflammatory cells such as macrophages infiltration are also increased in adipose tissues [12]. Proinflammatory cytokines and oxidative stress have also been shown to be responsible for developing metabolic disturbances, such as insulin resistance and activation of immune response in liver, adipose tissue, and muscle [13–15]. Moreover, activation of inflammatory pathways in hepatocytes is sufficient to cause both local as well as systemic insulin resistance [16, 17].

In the last decade, much attention has been focused on several molecular drug targets with the potential to prevent or treat metabolic disorders. Thus, nuclear receptors and their regulators have attracted much attention due to their regulatory role in both glucose homeostasis and lipogenesis [18]. Peroxisome proliferator-activated receptors (PPARs) and liver X receptors (LXRs) are two regulatory proteins identified to play a pivotal role in the regulation of metabolic homeostasis [19–21], while PPARs activation is important for lipid metabolism, adipocyte differentiation, and the prevention of inflammation [22]. PPARs also regulate mitochondrial biogenesis via an activator called PGC-1*α* [23, 24] which is physiologically regulated by exercise [25, 26] and calorie restriction [27]. In addition to these factors, pharmacological agents such as fenofibrates [28] and resveratrol [29] may also stimulate PGC-1*α* and restore mitochondrial function. Recent reports suggest that natural products are rich source of ligands for nuclear receptors and are promising therapeutic agents in clinical practice. Researchers have also examined the effects of various functional foods on overall body composition and selective fat depots. Water-soluble extract of* Cucurbita moschata* stem (500 mg/kg/day for 8 weeks) activated PPAR-*α*, increased *β*-oxidation, and inhibited adipocyte differentiation in a dose dependent manner [30]. Extracts of* Euonymus alatus* increased the expression of PPAR-*γ* in periepididymal fat pad and ameliorated the hyperglycemia and hyperlipidemia induced by high-fat diet* in vivo *[31]. Acacia polyphenols increased the mRNA expression of fat metabolizing genes like PPAR-*α* and PPAR-*δ* in skeletal muscle and lowered the expression of fat acid synthesis-related genes (SREBP-1c, ACC, and FAS) in the liver [32]. Green tea catechins have also been proposed as therapeutic agents for body fat reduction [33].

Bitter melon (*Momordica charantia *L.) is widely used for the treatment of diabetes. Recent research reports suggest that bitter melon extracts may ameliorate high fat diet induced obesity and hyperlipidemia in animal model. Most findings related to obesity and hyperlipidemia also showed that the plant extracts may modulate fat metabolizing kinases such as AMPKs, genes, and nuclear factors like PPARs, LXRs, and PGC-1*α*, in liver and skeletal muscle and affected adipocyte differentiation, while several review papers suggest the antidiabetic mechanism [34] and various pharmaceutical effects of the plant [35] and emphasized its efficacy and safety aspects. Therefore, the present review aims to describe the effect of bitter melon extracts on various parameters of metabolic syndrome, obesity, and related cardiovascular complications. Moreover, this review will also find out the plausible mechanisms responsible for antiobesity and hypolipidemic effects of the plant based on available information to date.

## 2. Bitter Melon Overview and Traditional Medicinal Uses

**Figure 1:**
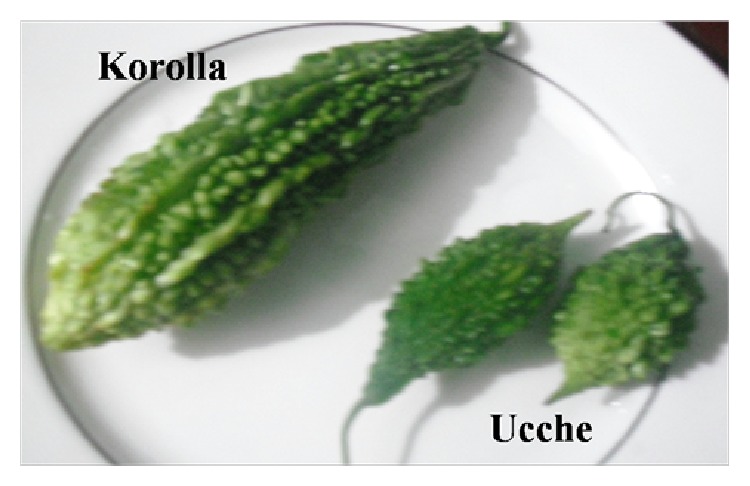
Fruits of different variety of* Momordica charantia* available in Bangladesh. Upper left one is commonly known as Korolla and right one as Ucche.

Bitter melon is a climbing shrub cultivated mainly in Bangladesh, India, China, and Korea, mostly in Asian countries. The plant also grows in tropical areas of Amazon, East Africa, and the Caribbean. It belongs to family* Cucurbitaceae* and the scientific name is* Momordica charantia*. Generally, two varieties of the plant are found in Bangladesh, while the small size one is locally called “*Ucche*” and the large size one is locally known as “*Korolla*” (Figure 1). However, some other wild type African species are also found in the country that include* M. balsamina* L.,* M. foetida* Schum., and* M. rostrata* A. Zimm. Bitter melon fruits are taken as culinary vegetable in Bangladesh and in Indian subcontinent; it is also used as a traditional medicinal plant for the treatment of various diseases in Bangladesh as well as other developing countries like Brazil, China, Colombia, Cuba, Ghana, Haiti, India Mexico, Malaya, Nicaragua, Panama, and Peru [35]. Perhaps the most common traditional use of the plant is to treat diabetes in different countries around the globe. It is also used for the treatment of various other pathological conditions such as dysmenorrhea, eczema, emmenagogue, galactagogue, gout, jaundice, kidney (stone), leprosy, leucorrhea, piles, pneumonia, psoriasis, rheumatism, and scabies [35].* Momordica charantia* are also documented to possess abortifacient, anthelmintic, contraceptive, antimalarial, and laxative properties [35].

Recent scientific exploration on this plant elucidated potential biological effect on both animal and clinical studies. Apart from its potential antibacterial [36] and antiviral activities [37], bitter melon extracts are also effective against cancer and were found to be effective for the treatment of ulcer, malaria, pain and inflammation, psoriasis, dyslipidemia, and hypertension.* Momordica charantia *also contains biologically active chemical compounds such as glycosides, saponins, alkaloids, fixed oils, triterpenes, proteins, and steroids [38]. Several other biologically active chemical constituents have so far been isolated from different parts of the plant, including the leaves, fruit pulp, and seeds.

*Typical Recipe of a Bitter Melon Dish Popular in Bangladesh*

*Bitter Melon Fry with Potato. *Ingredients are as follows:

 bitter melon (finely chopped): 100 g,
 potato (finely chopped): 1-2 (whole potato),
 onion: 1 full (finely chopped),
 garlic paste: half table spoon,
 hot chilli: 2 pieces,
 curcuma powder: half table spoon,
 oil (soyabean): 1-2 table spoon full,
 salt: (q.s.t).

First fry the chopped onion, garlic, and chilli together with soyabean oil in a cocking pan. Add some curcuma powder and salt and fry gently. After finishing this stage, add chopped bitter melon and potato in fried onion and fry until the potato and melon get brown color on its surface and a nice smell will come out from the dish. Serve it with warm rice or paratha bread made up of flour, having layers and fried in vegetable oil.

## 3. Chemical Constituents Isolated from Bitter Melon

*M. charantia* contains a number of chemical substances including nutritionally important vitamins, minerals, antioxidants, and many other phytochemicals, that is, glycosides, saponins, phenolic constituents, fixed oils, alkaloids, reducing sugars, resins, and free acids [35]. The immature fruits are also good source of vitamin C and also provide vitamin A, phosphorus, and iron [35]. Depending on the characteristics nature of the isolated compounds, they can be divided into several groups such as phenolic and flavonoid compounds, cucurbitane type triterpenoids, cucurbitane type triterpene glycoside, oleanane type triterpene saponins, and insulin like peptides.

### 3.1. Phenolic and Flavonoids Compounds

**Figure 2:**
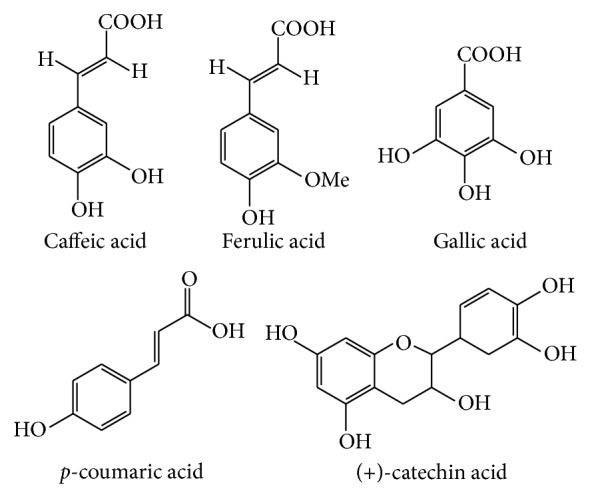
Different phenolic compounds isolated from* M. charantia*.

Phenolic compounds isolated from* M. charantia* are gallic acid, tannic acid, (+)-catechin, caffeic acid,* p*-coumaric, gentisic acid, chlorogenic acid, and epicatechin [39–41].  Figure 2 illustrates the phenolic constituents which have been isolated from* M. charantia* using high performance liquid chromatography (HPLC) analysis.

### 3.2. Cucurbitane Type Triterpenoids

**Figure 3:**
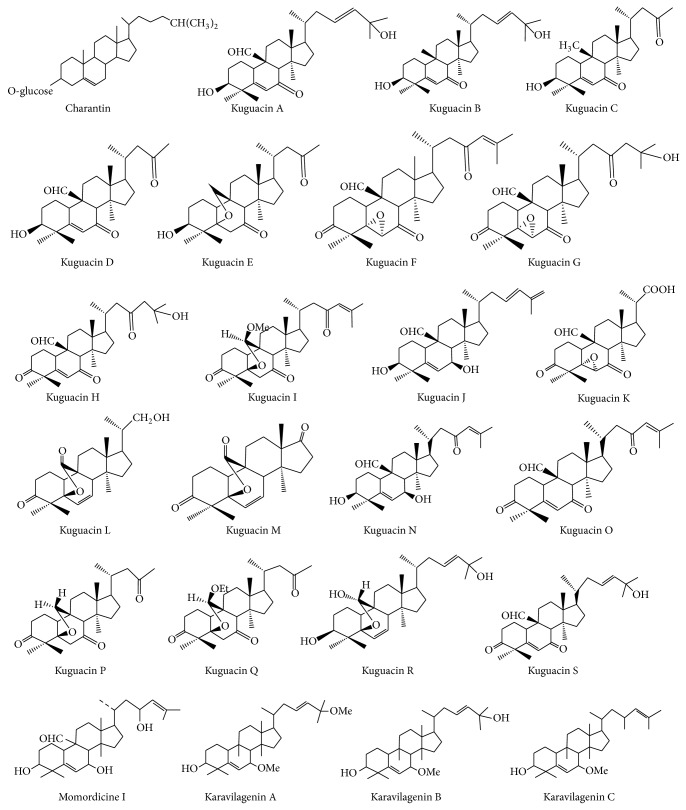
Chemical structure of some cucurbitane triterpenoids isolated from* M. charantia*.

The terpenoids are isoprenoids derived from five carbon isoprene units. The cucurbitacins are a typical group of cucurbitane type triterpenoids found mainly in cucumber family (Cucurbitaceae). The main chemical constituents of* M. charantia* are cucurbitane type triterpenoids [42–44] including charantin [45], different kuguacins [46], momordicin, and karavilagenins [47].  Figure 3 represents the chemical structures of the triterpenoids found in the plant.

### 3.3. Cucurbitane Type Triterpene Glycoside

Cucurbitane glycosides isolated from* M. charantia* are charantosides I–VIII [48]; momordicosides F1, F2, G, I, K, L, M, N, O, Q, R, S, and T [49–51]; karavilosides I, II, III, IV, V, VI, VII, VIII, IX, X, and XI [47]. Other cucurbitane type triterpene glycosides include 3-O-*β*-D-allopyranosyl, 7*β*, 25-dihydroxycucurbita-5, and 23(E)-diene-19-al [52]; 3-O-*β*-D-allopyranosyl, 7*β*, 25-dihydroxy cucurbita-5(6), 23(E)-diene-19-al, 3-O-*β*-D-allo pyranosyl, 25-methoxy cucurbita-5(6), and 23(E)- diene-19-ol [53]; goyaglycoside-a, -b, -c, -d, -e, -f, -g, and -h [54]. Cao et al. recently isolated and identified new cucurbitane triterpenes, 5*β*,19-epoxy-cucurbita-6,22*E*,24-trien-3*β*-ol, from acid-treated ethanol extract of the plant [55].

**Figure 4:**
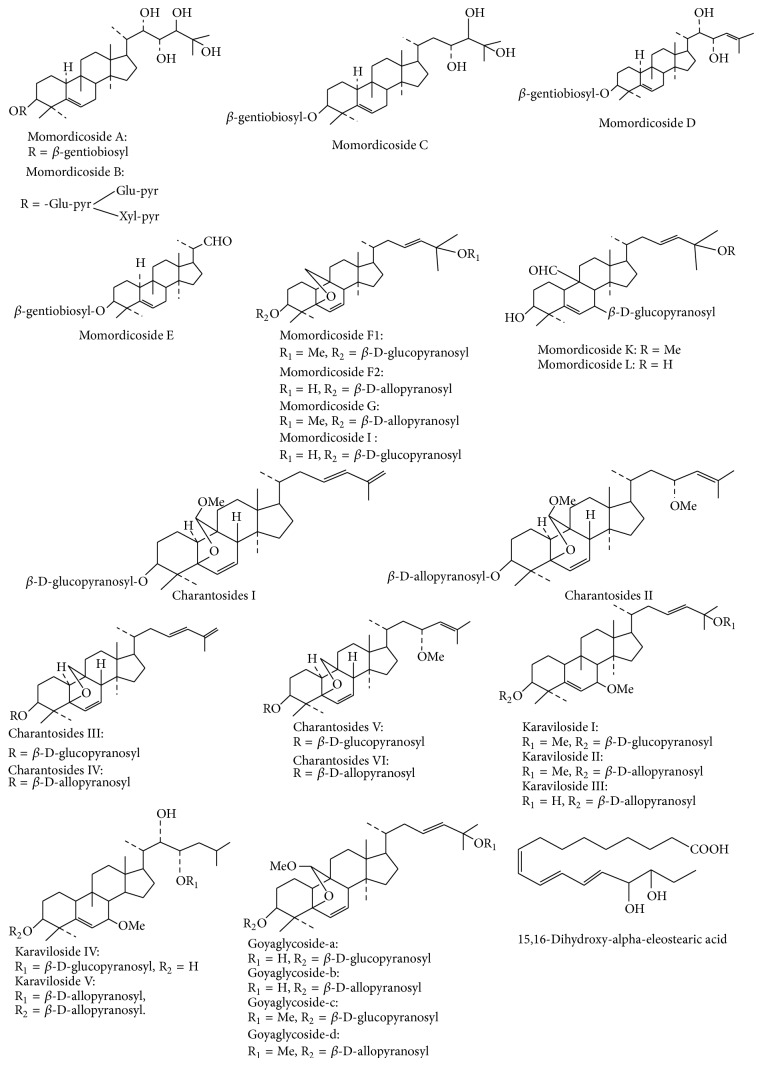
Chemical structure of major cucurbitane glycosides isolated from* M. charantia*.

Cucurbitane type triterpinoid glycosides, which are abundantly present in Momordica genus and have been isolated from* M. charantia,* are presented in Figure 4.

### 3.4. Oleanane Type Triterpene Saponins

Several oleanane type triterpene saponins such as soyasaponins I, II, and III have been isolated from the fresh fruit of Japanese* M. Charantia* [56].

### 3.5. Peptides

Recently two novel peptides, MCh-1 and MCh-2, have been isolated from the seeds of bitter melon [57].

## 4. Effect of Bitter Melon on Body Weight, Obesity, and Adipocyte Dysfunction

**Figure 5:**
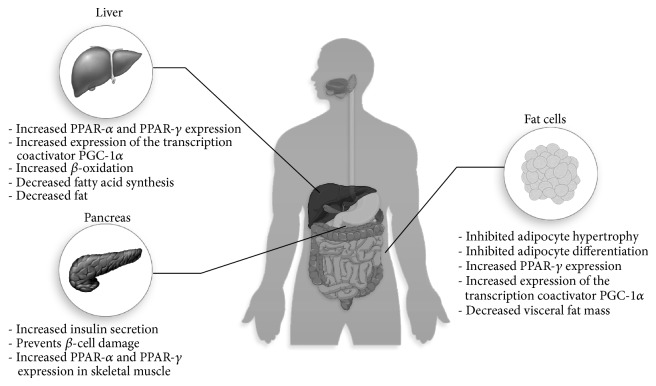
Effect of bitter melon on various organ and probable molecular targets for improving obesity and diabetes.

Body weight gain and abdominal fat deposition are the early signs of obesity. Bitter melon extract showed useful benefit on body weight gain and fat deposition (Figure 5, Table 1). Several investigational reports suggest that bitter melon can reduce body weight in high fat diet induced obesity in laboratory animals. Bitter melon (0.75% of diet) supplementation prevented the body weight gain and visceral fat mass significantly in rats fed high fat diet [58]. This weight reduction may be a result of increased fatty acid oxidation which ultimately facilitates weight reduction [58]. Moreover, the bitter melon extract supplementation reduced the peritoneal fat deposition in rats fed a high fat diet [58]. In another study, bitter melon significantly decreased the weights of epididymal white adipose tissue (WAT), visceral fat, and the adipose leptin and resistin mRNA levels in C57BL/6J mice fed with a high-fat (HF) diet [59]. Bano et al. reported that 2 mL/day dose of aqueous extract of bitter melon significantly reduced body weight gain in rats [60]. A recent study also showed that the seed oil supplementation of the plant reduced body weight and fat mass in mice fed a high fat diet [61].

Several mechanisms for lowering fat mass in obesity have been proposed. Generally, increased fatty acid transport would facilitate fat burning in tissues. Carnitine palmitoyltransferase (CPT) system is the predominant system for transporting the fatty acid to mitochondrial matrix [83]. Two CPTs were identified so far, CPT-1 and CPT-2, and a carnitine. CPT-1 resides on the inner surface of the outer mitochondrial membrane and is a major site of regulation of mitochondrial fatty acid uptake. It is evident that obesity may reduce the lipid oxidation in skeletal muscle due to the reduced expression and activity of CPT system in human and animal [84]. Earlier investigations also suggest that inhibition of CPT-1 with the chemical etomoxir increases lipid deposition and exacerbates insulin resistance when animals are placed on a HF diet [85], whereas overexpression of CPT-1 improved lipid-induced insulin resistance [86]. Additionally, increased skeletal muscle CPT-1 protein expression is sufficient to increase fatty acid oxidation and to prevent HF diet-induced fatty acid esterification into intracellular lipids, subsequently leading to enhanced muscle insulin sensitivity in HF-fed rats [87]. Bitter melon supplementation in these rats significantly decreased the body weight gain by increasing the hepatic and muscle mitochondrial carnitine palmitoyltransferase-I (CPT-1) and acyl-CoA dehydrogenase enzyme [62].

Mitochondrial uncoupling is another process in mitochondria whereby most of the energy consumed will be converted into heat rather than producing ATP. The proton gradient generated for the ATP synthesis is consumed through specified protein function known as uncoupling proteins which are attaining interest in recent years because of their critical role in energy expenditure and lipid metabolism [88]. Several uncoupling proteins have been isolated, that is, UCP1, UCP2, UCP3, UCP4, and UCP5. These proteins are distinctively expressed in several tissues and primarily participate in proton leaking. Alteration in function of these proteins will be beneficial in weight reduction in obesity [88]. In mice, genetic manipulation of UCP3 in skeletal muscle suggests that this protein is involved in the regulation of energy expenditure [89]. UCP1 in brown adipose tissue (BAT) and UCP3 in red gastrocnemius muscle were increased due to bitter melon supplementation followed by increased expression of the transcription co-activator PGC-1*α*, a key regulator of lipid oxidation [62].

Adipose tissues also play a central role in obesity. Bitter melon supplementation prevented the adipocyte hypertrophy in rats fed HF diet [63]. Its supplementation suppressed the visceral fat accumulation and inhibited adipocyte hypertrophy probably by lowering mRNA levels of fatty acid synthase, acetyl-CoA carboxylase-1, lipoprotein lipase, and adipocyte fatty acid-binding protein, downregulating lipogenic genes in adipose tissues [63]. A recent study suggests that bitter melon seed oil may increase the adipocyte death by cAMP-activated protein kinase (PKA) mediated apoptosis in white adipose tissues (WAT) of HF diet fed mice [61]. Previous* in vitro* study also showed prevention of preadipocyte differentiation and lipid accumulation in adipocyte by the plant extract. Bitter melon treatment of 3T3-L1 cells resulted in a decreased lipid accumulation and significantly decreased intracellular triglyceride (TG) amount compared to untreated control cells [64]. Moreover, bitter melon reduced the lipid accumulation during differentiation from a preadipocyte to adipocyte and downregulated PPAR*γ* [64]. PPAR*γ* is considered the master regulator of adipogenesis during differentiation of preadipocyte to adipocyte [90]. Other adipogenic transcription factors include the CCAAT/enhancer binding proteins (C/EBP*α*, C/EBP*β*, and C/EBP*δ*) and sterol regulatory element-binding protein 1c (SREBP-1c) [91]. Bitter melon juice inhibited adipocyte differentiation by reducing PPAR*γ*, SREBP, and perilipin mRNA gene expression and by increasing lipolysis in primary human adipocyte [65].

## 5. Effect of Bitter Melon on Dyslipidemia

Dyslipidemia are disorders related to increased cholesterol synthesis and abnormal lipoprotein metabolism, including lipoprotein overproduction and deficiency which are the early manifestations of obesity. Plasma lipids such as cholesterol, fatty acids, and TG concentrations are increased due to diabetes and HF diet feeding in laboratory animal and human [92]. Dyslipidemia is widely accepted as independent risk factor for coronary heart disease and associated with insulin resistance in type 2 diabetes mellitus [93]. The main cause of increased cholesterol and TGs in diabetic dyslipidemia is the increased FFA release from insulin-resistant fat cells [94]. Thus, FFAs overload into the liver and increased glycogen stores promote TG production, which in turn stimulates the secretion of apolipoprotein B (ApoB) and very low-density lipoprotein (VLDL) cholesterol [93, 94]. The hepatic overproduction of VLDL appears to be the primary and crucial defect of the insulin resistant accompanying obesity.

Bitter melon extracts showed lipid lowering effect both in diabetic and HF diet fed rats (Table 2). Bitter melon exhibited a marked reduction in the hepatic TC and TG in dietary cholesterol fed rats [66]. However, the bitter melon extract showed little effect on serum lipid parameters but increased HDL-C both in the presence and absence of dietary cholesterol in rats [66]. Ahmed et al. reported that 10 weeks of supplementation of the plant extract normalized the increased plasma nonesterified cholesterol, TGs, and phospholipids in streptozotocin- (STZ-) induced diabetic rats [67]. Treatment for 30 days with* Momordica charantia* fruit extract to diabetic rats also decreased TG and LDL and increased HDL level significantly [68]. Chen and Li also reported that 0.75% bitter melon extracts supplementation reduced the plasma cholesterol in rats fed a HF diet [58]. Another study showed that bitter melon reduced TG and LDL levels and increased HDL levels in high sucrose fed rats [71]. Ground bitter melon seeds (3.0% wt/wt) decreased TC and LDL-C and increased HDL-C in female Zucker rats [73]. The plant supplementation also decreased plasma level of TG, cholesterol, and FFA in plasma of offspring rats fed a HF diet [72]. Oishi et al. reported that saponin fraction of the plant decreased the TAG and pancreatic lipase activity in corn oil loaded rats [69]. Decreased pancreatic lipase activity is particularly important in fat absorption from gut wall as it enhances the fat digestion to fatty acids and increased plasma fatty acid level after fat intake. Thus reduction of pancreatic lipase would be a crucial target for lowering circulating FFAs.

The molecular mechanisms behind the lipid lowering effect of bitter melon extracts are revealed only recently. Freeze-dried bitter melon juice (1.5%) with diet normalized plasma TAG, cholesterol, and NEFA in female C57BL/6 mice fed a HF diet [70]. In this study, the juice in diet also decreased ApoB secretion and modulated the phosphorylation status of insulin receptor (IR) and its downstream signalling molecules [70]. Insulin resistance and dyslipidemia are characterized by significant downregulation of hepatic insulin signalling as documented by attenuated phosphorylation of IR and IR substrates (IRS) [95]. A direct link between attenuated hepatic insulin signalling and synthesis and secretion of VLDL apoB was established before [96]. VLDL particles are mainly cleared from circulation by the LDL receptor (LDLR), also referred to as apoB/E receptor. The transcription of the LDLR gene is regulated by intracellular cholesterol concentration, hormones, and growth factors. Moreover, sterol regulatory element binding protein-1 (SREBP-1) is selectively involved in the signal transduction pathway of insulin and insulin-like growth factor-I (IGF-I) leading to LDLR gene activation contributing to the delayed VLDL particle clearance associated with obesity causing insulin resistance [97]. Transcription factors in the SREBP family are key regulators of the lipogenic genes in the liver.

Increased mitochondrial biogenesis would be a possible way of increasing lipid metabolism and utilization in energy demanding cells and tissues. Mitochondrial biogenesis is regulated via several transcriptional regulatory factors like AMPK, PPAR-*γ*, and PGC-1*α* [98, 99]. AMPK regulated PPAR-*γ* and PGC-1*α* activation stimulated most of the transcriptional signal to increase fatty acid oxidation and mitochondrial function [100–102]. Bitter melon supplementation increased PPAR*γ* coactivator 1-*α* (PGC 1 *α*) and fibroblast growth factor 21 mRNA and fatty acid binding protein 1 in offspring of HF diet fed rats [72]. PGC-1 family of coactivator is of particular importance in the control of liver metabolism [98]. PGC-1*α* stimulates mitochondrial biogenesis and respiration in multiple cell types and modulates biological programs normally associated with increased oxidative metabolism [103]. This PGC-1*α* activation and upregulation by bitter melon supplementation also decreased plasma level of TGs, cholesterol, and FFA in plasma of offspring rats fed a HF diet [72]. Recent investigation also reported that 1.2% bitter melon extract supplementation significantly increased hepatic AMPK p, AMPK *α*1, AMPK *α*2, and Sirt1 content in HF diet fed mice [74]. AMP-activated protein kinase (AMPK) is a cellular fuel gauge, maintaining intracellular energy balance in mammalian cells [104]. AMPK activation is necessary for the transcriptional regulation of energy demand. Mice expressing a dominant-negative form of AMPK failed to increase mitochondrial biogenesis in response to energy deprivation in skeletal muscles [105]. In contrast, lipid oxidation and mitochondrial activity were increased in mice over expressing the phosphorylated AMPK [106, 107]. Thus, AMPK activation followed by Sirt1 due to the plant extract supplementation decreased TC, TGs, and LDL-Cin HF diet fed mice [74]. Bitter melon extract supplementation also decreased serum TC and fatty acids in C57BL/6J mice 45% high-fat (HF) diet [76]. This lipid lowering effect is attributed to its ability to increase AMPK phosphorylation and PPAR*γ* mediated lipid metabolism in liver [76].

The plant extract supplementation also decreased mRNA levels of hepatic LXR*α* and increased the hepatic CYP7A1 mRNA level in rats [75]. LXRs were first identified as orphan members of the nuclear receptor super family and oxidized derivatives of cholesterol act as ligands for the LXRs. LXR also plays an important role in lipid and cholesterol metabolism. LXR*α* knockout mice develop enlarged fatty livers, degeneration of liver cells, high cholesterol levels in liver, and impaired liver function when fed a high-cholesterol diet [108]. Hepatic LXR*α* downregulation due to bitter melon extract supplementation also decreased serum TC and LDL-C HDL-C in Wistar rats fed high cholesterol diet [75].

## 6. Effect of Bitter Melon on Nonalcoholic Fatty Liver and Liver Diseases

Hepatoprotective effect of bitter melon extracts is mainly attributed to its antioxidant capacity to scavenge free radicals and reduced inflammation in liver due to noxious stimuli. Chaudhari et al. reported that hydroalcoholic extract of the plant leaves (100 and 200 mg/kg) normalized the levels of ALT, AST, ALP, and total bilirubin and prevented steatosis, centrilobular necrosis, and vacuolization in liver of carbon tetrachloride induced liver damage in rats [109]. ALT, AST, and ALP are liver enzymes that significantly increased due to increased metabolism or damage to the liver tissues. The study by Thenmozhi and Subramanian also confirmed the antioxidant and hepatoprotective potential of its fruit extract in ammonium chloride-induced toxicity in rats [110]. Fruit extract (300 mg/kg) of the plant normalized the elevated TBARS, hydroperoxides, and liver markers (alanine transaminase, ALT; aspartate transaminase, AST; and alkaline phosphatase, ALP) and increased the levels of glutathione peroxidase (GPx), superoxide dismutase (SOD), and catalase and reduced glutathione in ammonium chloride-induced toxicity in rats [110]. The plant extract at a dose of 5 mL/kg also produced significant protection of liver damage due to high dose of acetaminophen administration in rabbits [111]. A recent study also suggests that bitter melon supplementation ameliorates oxidative stress in liver of fructose fed offspring of rats by improving the antioxidant enzymes activity such as GPx, SOD, and catalase [112].

Liver is the first line organ which undergoes direct challenges during diet induced obesity and diabetes. Excess fat intake overwhelms the hepatic tissues to metabolize them and undergoes fatty acid mediated inflammation and oxidative stress [113]. Excess fat accumulation in liver can be a result of one or a combination of the following metabolic alterations: (a) decreased *β*-oxidation of fatty acids, (b) increased fatty acid synthesis due to up-regulation of lipogenic pathways, (c) increased delivery of fatty acids from adipose and other organs due to lipolysis, and (d) inhibition of VLDL-TG export [114]. Numerous studies indicated that high fat and fructose overconsumption leads to the development of metabolic syndrome, including insulin resistance, dyslipidemia, and hypertension in humans [115, 116] and animals [113, 117]. High fat diet also develops hepatic steatosis in animal by accumulating lipid in hepatic tissues [113]. Bitter melon supplementation reduced the fat accumulation in liver and prevented steatosis in mice fed a high fat diet [74]. In this mice model, high fat diet feeding caused upregulation of fibroblast growth factor 21 levels in liver [74]. FGF family plays valuable role in the development of NAFLD [118]. It has been shown that plasma FGF21 levels were increased in patients with insulin resistant type 2 diabetes mellitus (T2DM) and in NAFLD patients who have high level of hepatic triglycerides (TG) [118, 119]. The plant supplementation downregulated hepatic FGF21 content significantly and increased hepatic phosphorylated AMPK, AMPK *α*1, AMPK *α*2, and Sirt1 content compared to the high fat diet fed mice [74].

## 7. Effect of Bitter Melon on Diabetes

Mostly reported biological activities of bitter melon are the effect on diabetes and hyperglycaemia. The plant showed potent antihyperglycemic effect in various animal models. An aqueous extract of bitter melon in normal mice lowered the glycaemic response to both oral and intraperitoneal glucose load without altering the insulin response [120]. Aqueous and alkaline chloroform extracts also reduced the hyperglycaemia in diabetic mice [120]. Pulp juice of this plant lowered fasting blood glucose and glucose intolerance in NIDDM model rats, while no effect was seen in STZ treated IDDM model rats [121]. In alloxan induced diabetic rats, blood sugar level was lowered after 3 weeks of treatment with aqueous extract of bitter melon fruits [122]. The plant extract also improved glucose intolerance in STZ treated diabetic rats and increased the glycogen synthesis in liver [123]. The aqueous extract powder of fresh unripe whole fruits at a dose of 20 mg/kg body weight reduced fasting blood glucose by 48% which is comparable to the effect of glibenclamide, a well-known oral antidiabetic drug, in rats [124]. Acute oral administrations of the whole plant extract also caused dose-related significant hypoglycaemia in normal (normoglycaemic) and STZ-treated diabetic rats [125].* M. charantia* extract also improved insulin sensitivity, glucose tolerance, and insulin signalling in high fat diet-induced insulin resistance rats [126].* M. charantia* also maintained the normal glucose concentration in chronic sucrose loaded rats [71].

Improvement of hyperglycaemic condition in experimental animal by* M. charantia* extracts has many plausible mechanisms, that is, (a) prevention of glucose absorption in the alimentary canal, (b) enhancing the glucose uptake by tissues, (c) increasing glucose metabolism, and (d) enhancing insulin like action and pancreatic beta cell stimulation [127]. Oral administration of the plant juice significantly reduced the Na^+^/K^+^ - dependent absorption of glucose from the intestinal mucosa in STZ-induced diabetic rats [128] which were also observed* in vitro* [129]. Moreover,* these* extracts may also inhibit carbohydrate metabolizing enzymes like alpha-amylase, alpha-glucosidase, and pancreatic lipase and hence limits the absorption of glucose through gut wall [130–132]. Several authors reported that the plant extract improves glucose uptake in cells, thereby increasing the glucose metabolism. Oral supplementation of the plant increased the muscle content of facilitative glucose transporter isoform 4 (GLUT4) proteins which might be responsible for significant improvement of oral glucose tolerance in KK-Ay mice, an animal model with type 2 diabetes with hyperinsulinemia [133]. Similar results were also reported by other investigators. Shih et al. reported that bitter melon extract significantly increases the mRNA expression and GLUT4 in skeletal muscle and normalized fructose diet-induced hyperglycemia in rats [134].

*In vitro* study suggested that the fresh juice of the plant increased the uptake of amino acids and glucose in L6 myotubes [135]. Aqueous and chloroform extracts of this fruit also increased glucose uptake and upregulated GLUT-4, PPAR-*γ*, and phosphatidylinositol-3 kinase (PI3K) in L6 myotubes [136]. The effects of the plant on glucose uptake and adiponectin secretion were also reported in adipose cells, 3T3-L1 adipocytes. Water-soluble components of the plant enhanced the glucose uptake at suboptimal concentrations of insulin in 3T3-L1 adipocytes [137].

*M. charantia* showed beneficial effect in diabetes by maintaining normal glucose levels and several investigators suggested that this beneficial effect is attributed to its ability to maintain the structural integrity of the pancreatic islets and also by regulating its functions like synthesis and release of hormones [138–140]. An investigation was carried out to observe the effect of* Momordica charantia* fruit juice on the distribution and number of *α*, *β*, and *δ* cells in the pancreas of STZ-induced diabetic rats and it was found that the juice significantly increased the number of *β* cells compared with untreated diabetic rats [138]. However, *α*-cells did not change significantly compared with untreated diabetic rats in this study. Oral administration of the seed extracts at a dosage of 150 mg/kg body weight for 30 days prevented degeneration of pancreatic islets and restored islets function [139]. Acetone extract of the plant fruit powder at doses 25, 50, and 100 mg/kg body weight affected different phases of recovery of *β*-cells of the islets of Langerhans and normalizes the functioning of the concerned cells [141]. Moreover, the fruit powder extracts enhanced neoformation of islets from preexisted islet cells along acinar tissues [141]. A recent report also suggests that administration of ethanolic extract of the fruit pulp of the plant in neonatal STZ-induced type 2 diabetic rats increased the islet size, total *β*-cell area, number of *β*-cells, and insulin levels compared with untreated diabetic rats [140].

Other scientists also reported insulin secretagogue properties of the plant as well. Subcutaneous administration of the protein extract isolated from* Momordica charantia* fruit pulp increased plasma insulin concentrations by 2-fold after 4 h of administration [142]. The fruit pulp protein extract also increased the insulin secretion but not glucagon in perfused rat pancreas [142]. A recent report also suggests that saponin significantly stimulated insulin secretion* in vitro* from pancreatic MIN6 *β*-cells [143].

Apart from these effects,* M. charantia* has also been reported to improve the sensitivity of insulin in hyperinsulinemia. Bitter melon supplementation improved insulin resistance and lowered serum insulin and leptin to the high fat diet fed rats [144]. It improves insulin sensitivity in skeletal muscle by increasing skeletal muscle insulin-stimulated IRS-1 tyrosine phosphorylation in high-fat-fed rats [126]. A recent report further confirmed that polypeptide isolated from the plant binds with IRs and modulates downstream insulin signalling pathways [145].

## 8. Effect of Bitter Melon on Hypertension and Vascular Dysfunction

Hypertension and vascular dysfunction are two metabolic disorders that occur during the progression of obesity and metabolic syndrome and most of the obese people are observed to develop moderate to high blood pressure. Whole-plant aqueous extract of the plant dose dependently normalized the hypertension in hypertensive Dahl salt-sensitive rats probably followed by acetylcholine mediated pathways [125]. A recent investigation also showed that* M. charantia *extracts lowered elevated blood pressure in Wistar rats after L-NAME challenge on 52 days [146]. Moreover, the plant extracts also reduced the angiotensin converting enzyme activities [146]. However, frustrating results were also observed in clinical setup. Recently, a preliminary open-label uncontrolled supplementation trial was conducted in 42 people with a mean age of 45.7 ± 11.4 years who were supplemented with 4.8 lyophilized encapsulated* Momordica charantia* powder daily for three months [147]. But no significant differences were noted in this study for high blood pressure, heart rate, and other parameters of metabolic syndrome [147].

## 9. Antioxidant Activity of Bitter Melon

Bitter melon showed potent antioxidant activities both* in vitro* and* in vivo* as several investigators reported the antioxidant activity of different parts of the plant from leaves to fruits. Aqueous and methanolic extracts of bitter melon showed increased metal chelating activity, prevented lipid peroxidation, and inhibited free radical generation in xanthine oxidase and cytochrome C mediated* in vitro* system [148]. Aqueous extracts of leaves of the plant significantly scavenge the DPPH free radicals and increased ferric reducing power, while fruits extracts scavenge hydroxyl radicals and showed increased total antioxidant capacity [39]. Phenolics extracted from pericarp and seed of the plant at three maturity stages also showed DPPH free radical scavenging activity [149]. Phenolic compounds isolated from the bitter melon are catechin, gallic acid, ferulic acid,* p*-coumaric acid, gentisic acid, chlorogenic acid, and epicatechin [39, 149].

The antioxidant activity of the total aqueous extract and total phenolic extract of* Momordica charantia* fruits was assessed in rat cardiac fibroblasts (RCFs), NIH 3T3, and keratinocyte (A431). No significant cytoprotection was observed with both the extracts used in H_2_O_2_ and xanthine oxidase induced damages in cells [150]. However, the plant extracts showed significant protection against oxidative stress in several* in vivo* models. Treatment with bitter melon extracts normalized the elevated concentrations of TBARS, hydroperoxides, and liver markers (ALP, AST, ALT) in hyperammonemic rats induced by ammonium chloride while reversing the oxidant-antioxidant imbalance [110]. This protective effect is mediated probably by increasing the activity and concentrations of GPx, SOD, and catalase and reduced glutathione in the liver and brain tissues [110]. The plant extracts also prevented the lipid peroxidation in chronic sucrose fed rats and normalized the reduced glutathione level in liver [71].

## 10. Clinical Studies That Used Bitter Melon

Bitter melon extracts are considered the most popular traditional medication used for the treatment of diabetes despite its bitter taste. Previous review report suggests that the clinical studies with bitter melon data with human subjects are limited and flawed by poor study design and low statistical power [151].  Table 3 summarized some important clinical studies that used various parts of bitter melon. Ahmad et al. reported that the aqueous homogenized suspension of the vegetable pulp significantly reduced both fasting and postprandial serum glucose levels in noninsulin dependent diabetic patients [77]. However, this study design was not a randomized placebo controlled study which lacks the appropriate comparison and biasness could not be excluded. Similar study was conducted by Tongia et al., who reported that 200 mg capsule of bitter melon twice daily synergistically improved hypoglycemic action of metformin and glibenclamide [78]. Randomized, double-blind, placebo-controlled trials with bitter melon are inconclusive and shortfall in appropriated study design, patient number, and duration of study. Fuangchan et al. reported a decline of fructosamine level in diabetic patients at week 4 with 2000 mg/day dose while other doses tested failed to show any significant effect [80]. Tsai et al. reported a decreased metabolic syndrome incidence rate compared to that at baseline and reduction of waist circumference in studied patients [81]. Trakoon-osot et al. also reported an improvement of diabetes condition in patients treated with bitter melon and a decline of advanced glycation end products (AGEs) in serum after 16 weeks of the intervention were reported [82]. However, other investigations reported by Dans et al. showed that two capsules of bitter melon three times a day after meals for 3 months failed to produce any significant improvement in diabetic conditions [79]. Almost all authors reported no serious side effects during the study period [152]. In some patients, headache, dizziness, stomach pain, and bloating were also reported.

## 11. Summary and Future Prospective

**Figure 6:**
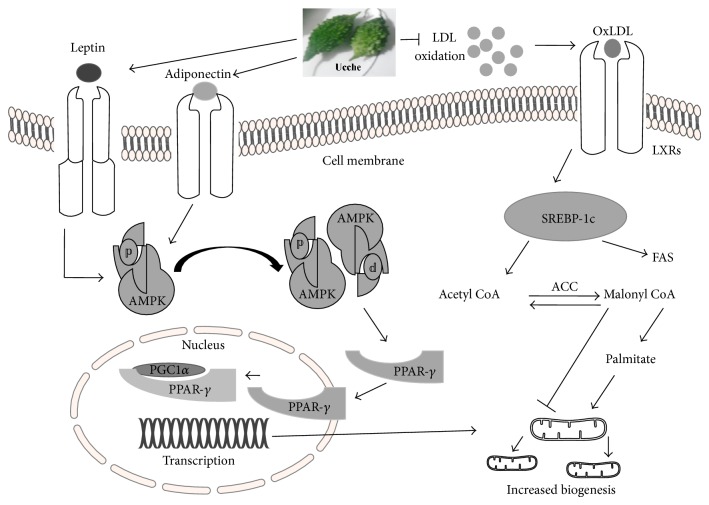
Hypothetical mechanism of bitter melon on fat metabolism in liver tissue via AMPK-PPAR*γ* mediated pathways.

To date*, M. charantia* has been extensively studied worldwide for its medicinal properties to treat a number of diseases like diabetes, dyslipidemia, obesity, and certain cancers. Isolated compounds from this plant like charantin, insulin-like peptide, and alkaloid-like extracts possess hypoglycemic properties similar to its crude extracts. The plant and fruit extracts and different compounds seem to exert their beneficial effects via several mechanisms like AMPK, PPARs, LXRs, SREBPs, Sirts mediated glucose, and fat metabolism in various tissues which are directly related to the beneficial effect of controlling and treating diabetes mellitus, dyslipidemia, and obesity related cardiovascular complications. A hypothetical mechanism has been proposed in Figure 6 which aimed to explain the lipid lowering effect of bitter melon. However, a knowledge gap in research was observed in the field of any direct effect of this plant on cardiac function, hypertension, and hypercholesterolemia induced atherosclerosis. Moreover, clinical studies reported mostly lack appropriate study design and are inconclusive. Thus, further studies are required to conduct more double blind randomized trials with bitter melon extracts in diabetes patients as well as in obese population. Further researches are also advocated for eliciting the effect of different dose of bitter melon in diabetic heart failure and hypertension both in animal and in patients with diabetes, obesity, and cardiovascular complications.

## Figures and Tables

**Table 1:** Effect of bitter melon on body weight, obesity, and adipocyte dysfunction.

Model	Dose	Experimental outcome	Reference
HF diet induced fat rats	0.75% and 1.5% extracts	(i) Decreased body weight, visceral fat mass, plasma glucose, and TAG. (ii) Increased plasma catecholamines.	[58]

HF diet induced fat rats	0.75% and 1.5% extracts	(i) Decreased body weight, visceral fat mass, plasma glucose, and TAG. (ii) Increased adiponectin. (iii) Increased UCP 1 in BAT and UCP 3 in red gastrocnemius muscle. (iv) Increased expression of the transcription coactivator PGC-1α both in BAT and in gastrocnemius muscle.	[62]

Male C57BL/6J mice, 5 weeks old	0.5 g/kg/day, 1.0 g/kg/day P extracts, or 0.2, 1.0 g/kg/day G extracts	(i) Decreased body weight and visceral fat mass. (ii) Decreased plasma glucose, TG, and total cholesterol (TC) but increased free fatty acid (FFA). (iii) Increased mRNA expression of leptin, PPAR-γ, PPAR-α, and decreased expression of resistin.	[59]

Male Wistar rats fed HF diet	5% (w/w) powder	(i) Decreased body weight and adipose tissues. (ii) Decreased TAG and cholesterol. (iii) Increased adiponectin.	[63]

Over weight rats	Aqueous extract 2 mL/day	(i) Reduced elevated body weight and cholesterol, TG, and low-density lipoprotein cholesterol (LDL-C). (ii) Increased high density lipoprotein cholesterol (HDL-C).	[60]

HF diet fed male C57BL/6JNarl mice.	15% and 30% of diet	(i) Decreased body weight, retroperitoneal, epididymal, and inguinal fat deposition and adipocyte diameter. (ii) Increased phosphorylation of acetyl-CoA carboxylase, cAMP-activated protein kinase (PKA), and signal transducer and activator of transcription 3 in WAT. (iii) Increased TNF-α concentration in the WAT accompanied by TUNEL-positive nuclei.	[61]

3T3-L1 cells		(i) Decreased lipid accumulation and intracellular TGs.	[64]

Primary human adipocyte		(i) Inhibited adipocyte differentiation by reducing PPARγ, SREBP, and perilipin mRNA gene expression. (ii) Increasing lipolysis in preadipocytes.	[65]

**Table 2:** Effect of bitter melon extracts on lipid parameters of diabetic and obese animal models.

Model	Dose	Experimental outcome	Reference
Cholesterol fed rats	0.5, 1 and 3% of diet	(i) Not changed TC level, but (ii) increased HDL-C level in plasma.	[66]

STZ-induced diabetic rats	10 mL 100% fruit extract per kg body weight daily for 10 weeks	(i) Decreased elevated level of plasma cholesterol, TGs and phospholipids in STZ induced diabetic rats.	[67]

Diabetic rats		(i) Decreased in TG and LDL, (ii) Increased in HDL.	[68]

Rats fed a HF diet	7.5 g/kg or 0.75%	(i) Supplementation did not affect serum and hepatic cholesterol. (ii) Supplementation in HF diet rats led to a lowering of hepatic TAG and steatosis score in liver section. (iii) Plasma epinephrine and serum FFA concentrations were increased. (iv) Lowered TAG concentration in red gastrocnemius and tibialis anterior.	[58]

Wistar rats	Saponin fraction (50–100 mg/kg body weight)	(i) Decreased pancreatic lipase activity and serum TG level in corn oil loaded rats.	[69]

Female C57BL/6 mice fed with HF diet	1.5% freeze-dried BMJ with diet	(i) Normalized plasma TAG, cholesterol, and NEFA. (ii) Normalized AST, ALT, and ALP in plasma. (iii) Decreased ApoB secretion and modulated the phosphorylation status of IR and its downstream signalling molecules.	[70]

Albino rats fed with sucrose	40, 80, and 120 mg/kg of body weight	(i) Reduced TG and LDL levels and increased HDL levels. (ii) Normalized hyperglycemia. (iii) Lowered TBARS and normalized levels of reduced glutathione.	[71]

Offspring rats fed high (60%) fructose diet	1% of diet	(i) Decreased plasma level of TG, cholesterol, and FFA. (ii) Lowered the hepatic levels of stearoyl-CoA desaturase and microsomal TG transfer protein mRNA. (iii) Increased PPARγ coactivator 1-α and fibroblast growth factor 21 mRNA and fatty acid binding protein 1.	[72]

Female Zucker rats	3.0% (wt = wt) ground BMS	(i) Supplementation increased the expression of PPAR-γ in the WAT. (ii) Decreased TC and LDL-C; increased HDL-C. (iii) Downregulated the expression of PPAR-γ, nuclear factor-kB (NF-kB), and interferon-γ mRNA in heart tissue.	[73]

HF diet fed mice	1.2% plant extract	(i) Decreased TC, TGs, and LDL-C. (ii) Increased hepatic AMPK p, AMPK α1 AMPK α2, and Sirt1 content. (iii) FGF21 and insulin concentrations were significantly decreased. (iv) Hepatic FGF21 content was significantly downregulated, while FGF receptors 1, 3, and 4 (FGFR1, FGFR3, and FGFR4) were greatly upregulated.	[74]

Wistar rats fed high cholesterol diet		(i) Decreased serum TC and LDL-C HDL-C. (ii) Decreased mRNA levels of hepatic LXRα in rats. (iii) Increased the hepatic CYP7A1 mRNA level.	[75]

C57BL/6J mice 45% HF diet	0.1, 0.2, and 0.4 g/kg/day extracts	(i) Decreased serum TC and fatty acids. (ii) Normalized leptin and insulin concentration. (iii) Increased PPARα level in liver. (iv) Increased GLUT4 expression in skeletal muscle. (v) Significantly increased the hepatic protein contents of AMPK phosphorylation and decreased expression of phosphoenolpyruvate carboxykinase (PEPCK).	[76]

**Table 3:** Clinical studies of bitter melon (MC).

Study design	Subject	Dose and duration	Outcome	Reference
Case study	100 moderate noninsulin dependent diabetic (NIDDM) subjects	Aqueous homogenized suspension of the vegetable pulp	Significant reduction of both fasting and postprandial serum glucose levels was observed	[77]

Case study	15 patients of either sex (52–65 years of age) of NIDDM	200 mg twice dailywith 7 days treatment plus half doses of metformin or glibenclamide or both in combination	The extract acts in synergism with oral hypoglycemics and potentiates their hypoglycemia in NIDDM	[78]

Randomized, double-blind, placebo-controlled trial	40 patients, 18 years old and above	Two capsules of *M. charantia* three times a day after meals, for 3 months	No significant effect on mean fasting blood sugar, total cholesterol, and weight or on serum creatinine, ALT, AST, sodium, and potassium	[79]

Multicenter, randomized, double-blind, active-control trial	The total of 143 patients were enrolled into the study; 129 patients were randomized to the either metformin (*n* = 33), bitter melon 500 mg/day (*n* = 33), bitter melon 1000 mg/day (*n* = 32), or bitter melon 2000 mg/day (*n* = 31)	Bitter melon 500 mg/day, 1000 mg/day, and 2000 mg/day or metformin 1000 mg/day for four weeks	2000 mg/day dose showed significant decline in fructosamine at week 4 500 and 1000 mg/day did not significantly decrease fructosamine levels	[80]

Open-label uncontrolled supplementation trial	42 eligible (21 men and 21 women) with a mean age of 45.7 ± 11.4 years (23 to 63 years)	4.8 gram lyophilized bitter melon powder in capsules daily for three months	The metabolic syndrome incidence rate decreased compared to that at baseline The waist circumference also significantly decreased after the supplementation	[81]

Two-arm, parallel, randomized, double-blinded, placebo-controlled trial	19 type 2 diabetic patients taken fruit pulp and 19 type 2 diabetic patients taken as placebo	6 g/day of MC dried-fruit pulp containing 6.26 ± 0.28 mg of charantin	Significant decline of total advanced glycation end-products (AGEs) in serum after 16 weeks of the intervention	[82]

## Abbreviations

ACC: Acetyl-CoA carboxylase
ACE: Angiotensin-converting enzyme
ALP: Alkaline phosphatase
AST: Aspartate transaminase
ALT: Alanine aminotransferase
AMPK: 5′ AMP-activated protein kinase (5′ adenosine monophosphate-activated protein kinase)
ApoB: Apolipoprotein B
BAT: Brown adipose tissue
BMI: Body mass index
C/EBP: CEBPB CCAAT/enhancer binding protein
cAMP: Cyclic adenosine monophosphate (3′-5′-cyclic adenosine monophosphate)
CPT-I: Carnitine palmitoyltransferase I
CYP7A1: Cholesterol 7 alpha-hydroxylase (cytochrome P450 7A1)
DPPH: 2,2-Diphenyl-1-picrylhydrazyl
FAS: Fatty acid synthase
FFA: Free fatty acid
FGF: Fibroblast growth factor
FGFR: Fibroblast growth factor receptor
GLUT4: Glucose transporter type 4
HDL: High-density lipoprotein
HDL-C: High-density lipoprotein cholesterol
HF: High fat
HPLC: High-performance liquid chromatography
IDDM: Insulin-dependent diabetes mellitus
IGF: Insulin-like growth factor
IL-6: Interleukin 6
IR: Insulin receptor
IRS-1: Insulin receptor substrate 1
LDL: Low-density lipoprotein
LDL-C: Low-density lipoprotein cholesterol
LDLR: Low density lipoprotein receptor
L-NAME: L-NG-nitroarginine methyl ester
LXR: Liver X receptors
MCP-1: Monocyte chemoattractant protein-1
mRNA: Messenger ribonucleic acid
NAFLD: Nonalcoholic fatty liver disease
NEFA: Nonesterified fatty acids
NF-*κ*B: Nuclear factor kappa-light-chain enhancer of activated B cells
NIDDM: Noninsulin-dependent diabetes mellitus
PEPCK: Phosphoenolpyruvate carboxykinase
PGC: Peroxisome proliferator-activated receptor gamma coactivator 1-alpha
PI3K: Phosphoinositide 3-kinase
PKA: Protein kinase A
PPAR: Peroxisome proliferator-activated receptor
RCF: Rat cardiac fibroblast
Sirt1: Sirtuin 1
SREBP: Sterol regulatory element-binding protein
STZ: Streptozotocin
T2DM: Type 2 diabetes mellitus
TAE: Total aqueous extract
TAG: Triacylglycerols
TBARS: Thiobarbituric acid reactive substances
TC: Total cholesterol
TG: Triglycerides
TNF: Tumor necrosis factor
VLDL: Very low-density lipoprotein
WAT: White adipose tissue
WHO: World Health Organization.
